# Buyang Huanwu Decoction protects against STZ-induced diabetic nephropathy by inhibiting TGF-β/Smad3 signaling-mediated renal fibrosis and inflammation

**DOI:** 10.1186/s13020-021-00531-1

**Published:** 2021-11-14

**Authors:** Weifeng Wu, Yifan Wang, Haidi Li, Haiyong Chen, Jiangang Shen

**Affiliations:** School of Chinese Medicine, Li Ka Shing Faculty of Medicine, The University of Hong Kong, Hong Kong, China

**Keywords:** Buyang Huanwu Decoction, Calycosin-7-Glucoside, Diabetic nephropathy, Fibrosis, Inflammation, TGF-β1, Smad3

## Abstract

**Background:**

Buyang Huanwu Decoction (BHD) is a classical Chinese Medicine formula empirically used for diabetic nephropathy (DN). However, its therapeutic efficacies and the underlying mechanisms remain obscure. In our study, we aim to evaluate the renoprotective effect of BHD on a streptozotocin (STZ)-induced diabetic nephropathy mouse model and explore the potential underlying mechanism in mouse mesangial cells (MCs) treated with high glucose in vitro, followed by screening the active compounds in BHD.

**Methods:**

Mice were received 50 mg/kg streptozotocin (STZ) or citrate buffer intraperitoneally for 5 consecutive days. BHD was intragastrically administrated for 12 weeks starting from week 4 after the diabetes induction. The quality control and quantitative analysis of BHD were studied by high-performance liquid chromatography (HPLC). Renal function was evaluated by urinary albumin excretion (UAE) using ELISA. The mesangial matrix expansion and renal fibrosis were measured using periodic acid-schiff (PAS) staining and Masson Trichrome staining. Mouse mesangial cells (MCs) were employed to study molecular mechanisms.

**Results:**

We found that the impaired renal function in diabetic nephropathy was significantly restored by BHD, as indicated by the decreased UAE without affecting the blood glucose level. Consistently, BHD markedly alleviated STZ-induced diabetic glomerulosclerosis and tubulointerstitial injury as shown by PAS staining, accompanied by a reduction of renal inflammation and fibrosis. Mechanistically, BHD inhibited the activation of TGF-β1/Smad3 and NF-κB signaling in diabetic nephropathy while suppressing Arkadia expression and restoring renal Smad7. We further found that calycosin-7-glucoside (CG) was one of the active compounds from BHD, which significantly suppressed high glucose-induced inflammation and fibrosis by inhibiting TGF-β1/Smad3 and NF-κB signaling pathways in mesangial cells.

**Conclusion:**

BHD could attenuate renal fibrosis and inflammation in STZ-induced diabetic kidneys via inhibiting TGF-β1/Smad3 and NF-κB signaling while suppressing the Arkadia and restoring renal Smad7. CG could be one of the active compounds in BHD to suppress renal inflammation and fibrosis in diabetic nephropathy.

**Supplementary Information:**

The online version contains supplementary material available at 10.1186/s13020-021-00531-1.

## Introduction

Diabetic nephropathy (DN) is the main cause of end-stage renal disease (ESRD) with high morbidity and mortality in diabetes mellitus (DM) patients [1]. In the United States, about 30–40% of diabetes cases develop into diabetic nephropathy. Diabetes mellitus accounts for 30–50% of total ESRD incidents, representing a significant public health concern [2]. Pathologically, diabetic nephropathy is characterized by chronic renal inflammation and fibrosis, leading to the deposition of extracellular matrix (ECM) in renal interstitial and thickening of basement membranes [3]. As no curative drug for diabetic nephropathy is available, most diabetic nephropathy patients eventually develop into ESRD.

In the progression of diabetic nephropathy, renal inflammation and fibrosis consistently appear, eventually leading to renal injury [3]. Multiple signaling pathways participate in the pathological process of renal inflammation and fibrosis during diabetic nephropathy. Among them, transforming growth factor β1 (TGF-β1) mediated Smad signaling is a representative signaling pathway contributing to diabetic nephropathy. TGF-β1 is abundantly expressed by all kinds of kidney resident cells and infiltrating inflammatory cells for the regulation of many signaling pathways, including both Smad-dependent and Smad-independent signaling [4]. Notably, TGF-β1/Smad3 signaling is involved in diabetic nephropathy [5, 6]. Smad3 knock-out mice or Smad3 inhibitors attenuate renal fibrosis and inflammation in diabetic mice, suggesting that the TGF-β1/Smad3 signaling pathway contributes to renal fibrosis and inflammation in diabetic nephropathy [7–9]. In contrast, an inhibitory Smad, Smad7, represses TGF-β/Smad3 and NF-κB signaling pathway by interacting with TGF-β receptors and functions as an antagonist of these molecules in diabetic nephropathy [10]. Overactivation of TGF-β1/Smad3 signaling is accompanied by the degradation of Smad7, contributing to the activation of NF-κB signaling in diabetic nephropathy [11]. Thus, suppression of TGF-β1/Smad3 signaling represents a critical therapeutic approach to reduce renal inflammation and fibrosis in diabetic nephropathy [9, 12, 13].

Traditional Chinese medicine (TCM) has a long history of treating various diseases with less toxicity and side effects [14, 15]. According to TCM concepts, the syndrome patterns of diabetic nephropathy can be classified as qi deficiency, blood stasis, yin deficiency, turbid dampness, phlegm dampness, yang deficiency, blood deficiency, and qi stagnation [16]. Based on clinical observation, qi deficiency-induced blood stasis is one of the most common TCM syndrome patterns in diabetic nephropathy patients. Thus, the herbal formula for tonifying qi to improve blood circulation has been commonly used for diabetic nephropathy treatment. Buyang Huanwu Decoction (BHD) is a classic TCM formula with the functions of replenishing qi and invigorating blood circulation, which is composed of *Astragali Radix*, *Angelicae Sinensis Radix Tail*, *Paeoniae Radix Rubra*, *Chuanxiong Rhizoma*, *Persicae Semen*, *Carthami Flos* and *Pheretima* with the ratio of 120:6:4.5:3:3:3:3 (dry weight) [17]. BHD is commonly used to treat diseases with the pathological status with Qi deficiency and blood stasis, such as vascular diseases, nerve injury-associated diseases, and kidney diseases in TCM practice [18, 19]. Our previous studies demonstrated that BHD has neuroprotective and neurogenesis-promoting effects in transient ischemic stroke model via activating PI3K/Akt/Bad and Jak2/Stat3/Cyclin D1 signaling pathways and modulating VEGF and Flk1 expressions [17, 20]. Recently, a meta-analysis consisting of 12 randomized controlled trials (RCTs) and involving 911 patients revealed that the BHD could be an adjunct therapy with RAAS inhibitors for early diabetic nephropathy patients by reducing the 24 h urine albumin excretion rate (UAER) [21]. BHD exerts anti-inflammation and anti-fibrotic bioactivities via distinct signaling pathways in several experimental in vivo models, such as transient focal cerebral ischemia [22], myocardial ischemia [23], and experimental autoimmune encephalomyelitis (EAE) [24]. However, whether BHD has anti-inflammation and anti-fibrosis effects and recovers renal function in diabetic nephropathy remains unknown. In the study, we tested the hypothesis that BHD could attenuate renal inflammation and fibrosis in diabetic nephropathy, and its underlying mechanisms would be related to inhibiting the TGF-β1/Smad3 pathway.

## Materials and methods

### Materials

Clinically used BHD concentrated Chinese Medicine Granules (CCMG) was obtained from PuraPharm International Ltd. (Hong Kong, China). Antibodies specific for fibronectin, collagen I, TNF-α and IL-1β were purchased from Abcam (Cambridge, UK). Antibodies for NF-κB p65, phosphor-NF-κB p-p65 and β-actin were purchased from Santa Cruz Biotechnology (Santa Cruz, CA, USA). Anti-Smad3 and anti-phospho-Smad3 (Ser423/425) antibodies were purchased from Cell Signaling Technology (CST, Danvers, MA, USA). Antibody anti-Smad7 was purchased from R&D Systems (R&D, Oxford, UK). Antibody for F4/80 was purchased from AbD Serotec (Oxford, UK). Anti-Arkadia antibody was purchased from Thermo Scientific (Waltham, MA, USA). Compounds for quality control analysis, including calycosin-7-glucoside, hydroxysafflor yellow A and amygdalin, were purchased from Nantong FeiYu biological technology Co., Ltd., China. Ferulic acid and paeoniflorin were purchased from Jiangsu Yongjian Pharmaceutical Co., Ltd., China. All standards were of purity greater than 98%.

### BHD and compounds solution

BHD concentrated Chinese Medicine Granules (CCMG) (PuraPharm Nongs) were used in our study. For qualitative and quantitative analyses, BHD was dissolved in water (100 mg/ml) under ultrasonic for 10 min at 35 ℃. According to Chinese Pharmacopoeia (2020 edition), the representative active chemical ingredients of *Astragali Radix*, *Angelicae Sinensis Radix Tail*, *Paeoniae Radix Rubra*, *Chuanxiong Rhizoma*, *Persicae Semen* and *Carthami Flos* are calycosin-7-glucoside, ferulic acid, paeoniflorin, ferulic acid, amygdalin and hydroxysafflor yellow A, respectively (Additional file 1: Table S1). The standard chemical ingredients calycosin-7-glucoside (0.05 mg/ml), hydroxysafflor yellow A (0.1 mg/ml), amygdalin (0.35 mg/ml), ferulic acid (0.1 mg/ml), paeoniflorin (0.35 mg/ml) were dissolved together in 50% methanol to prepare a mixed standard working solution.

### Quality control analysis

We performed the HPLC fingerprint study on BHD. Chromatographic separations were operated on a Thermo UPLC system with ACE Excel 2 C18 column (100 × 2.1 mm, 2 μm). The following chromatographic parameters were optimized in the study: mobile phase A (acetonitrile) and mobile phase B (0.1% formic acid–water), the flow rate at 0.4 ml/min, column temperature at 25 ℃, and detection wavelength at 227 nm. The whole retention time was 50 min. The percentage of mobile phase A increased from 1 to 10% during the first 10 min, then the ratio of two mobile phases was held for the next 38 min before the percentage of phase A raised to 15% in the last 2 min.

### Quantitative quality control analysis

Five standard chemical ingredients were used to perform quantitative analysis. After obtaining the standard curve of each chemical ingredient, mixed standard working solutions were analyzed by HPLC under the same condition as the Quality Control Analysis of BHD. The measurement was conducted 3 times parallelly. According to the standard curve, the content of each standard chemical ingredient was calculated.

### Animals and STZ-induced diabetic nephropathy mouse model

Male ICR mice (10–12 weeks old) were supplied from Laboratory Animal Unit at the University of Hong Kong. All experimental protocols of animals were approved by the Committee on the Use of Live Animals in Teaching and Research (CULATR). The mice were maintained on 12-h light/dark cycles in a pathogen-free environment with a constant temperature of 22 ℃. The type I diabetes model was induced in mice according to the low-dose STZ induction protocol recommended by the Animal Models of Diabetic Complications Consortium (https://www.diacomp.org/). Streptozotocin (STZ, S0130, Sigma-Aldrich Corp, St. Louis, MO, USA) was freshly prepared in 0.1 M sodium citrate buffer (pH 4.5). Mice were fasted for 4 h before STZ administration. After 4 h fasting, mice were received a daily intraperitoneal injection of 50 mg/kg STZ or sodium citrate buffer for 5 consecutive days. Fasting blood glucose and urine were collected every 2 or 4 weeks. The mice were sacrificed at week 16 after STZ injection. Kidney tissues were collected and stored at – 80 ºC or paraffin-embedded for subsequent experiments.

### Microalbumin and renal function

Renal function was evaluated by urinary albumin excretion (UAE), the ratio of total urinary albumin/creatinine. For urine analysis, 24 h urine samples were collected from metabolic cages every 2–4 weeks. Microalbuminuria was quantified using the competitive ELISA method (Exocell, Philadelphia, PA, USA) and creatinine was detected by the Creatinine Companion kit (Exocell, Philadelphia, PA, USA) according to the manufacturers' instructions.

### Renal histology and immunohistochemistry

The kidney tissues were fixed with 4% paraformaldehyde (PFA) and embedded in paraffin, then cut into 5 μm sections as previously described [10]. Mesangial matrix expansion was measured by Periodic acid Schiff (PAS) staining, which was scored by determining the percentage of tubules that displayed tubular necrosis, cast formation, and tubular dilation as follows: 0  =  normal; 1  =  1–10%; 2  =  10–25%; 3  =  26–50%; 4  =  51–75%; 5  =  75–95%; 6  =   > 96% [25]. Masson Trichrome staining was performed to determine renal fibrosis resulting from extracellular matrix deposition (ECM) by the NovaUltraTM Masson Trichrome Staining kit (IHC World, Woodstock, MD). ECM was examined by ten random view fields (at 400 ×) for each kidney. The immunohistochemical staining for F4/80, fibronectin, IL-1β, phospho-Smad3, phospho-p65 were conducted as described previously [3].

### Cell culture and drug treatments

Mouse mesangial cells (MCs) were used in our study. Cells were grown in Dulbecco’s modified Eagle’s medium (DMEM)/Ham's F12 medium (Invitrogen Life Technologies, Carlsbad, CA, USA) supplemented with 10% fetal bovine serum (FBS; Invitrogen life Technologies) and 1% penicillin/streptomycin (Gibco) at 37 °C in a humidified atmosphere with 5% CO_2_. The MCs were cultured in an FBS-free medium for 24 h and then stimulated with BHD or compounds under normal D-glucose (5.5 mM) or high D-glucose (35 mM) conditions for up to 24 h. D-Mannitol (29.5 mM) was used as an osmotic control. MCs were pre-treated with 2 μM SIS3 for 1 h before stimulating with high glucose for positive control groups. Cells were harvested for western blot and real-time PCR analysis. All experiments were conducted three or four times.

### BHD treatments in animals

Mice orally received BHD at 0.5 g/kg, 1 g/kg and 2 g/kg daily for 12 weeks from week 4 after STZ injection. Irbesartan (IRB) (50 mg/kg per day) was taken as a positive control.

### RNA extraction and RT-qPCR

Total RNA was isolated from harvested cells using an RNA extraction kit (RNeasy; Qiagen, Valencia, CA, USA). Real-time PCR was performed using the Bio-Rad iQ SYBR Green supermix with Opticon 2 (Bio-Rad, Hercules, CA, USA) as previously described [3, 10]. Fibronectin (FN) and IL-1β were analyzed by RT-qPCR analysis. β-actin was used as an internal control. The primers used in this study are listed as follows:

Mouse Fibronectin, forward: 5′-TACCAAGGTCAATCCACACCCC-3′, reverse: 5′-CAGATGGCAAAAGAAAGCAGAGG-3′

Mouse IL-1β, forward: 5′-CTTCAGGCAGGCAGTATCACTCAT-3′, reverse: 5′-TCTAATGGGAACGTCACACACCAG-3′

### Western blot analysis

Protein was extracted from renal tissues or cells by RIPA buffer, and western blot analysis was carried out as described previously [3]. Briefly, membranes were incubated with primary antibodies against fibronectin, collagen I, TNF-α, IL-1β, NF-κB p65, phospho-NF-κB p-p65, Smad3, phospho-Smad3 (Ser423/425), Smad7, Arkadia and β-actin at 4 °C overnight, and then the membranes were further incubated in HRP conjugated second antibodies. The signals of proteins were detected by chemiluminescent ECL™ Detection Kit (GE Healthcare), followed by scanning using Gel Doc system (Bio-Rad) and analyzing by Image Lab software (Bio-Rad). Protein levels were quantified using the ImageJ software (NIH, Bethesda, MA, USA).

### Statistical analysis

Data are shown as the mean  ±  SEM and were analyzed using one-way analysis of variance (ANOVA), followed by Tukey’s post-hoc tests using GraphPad Prism Version 7.0 software (GraphPad Software Inc., CA, USA). A value of p  <  0.05 was considered statistically significant.

## Results

### HPLC analysis for quality control study of BHD

**Fig. 1:**
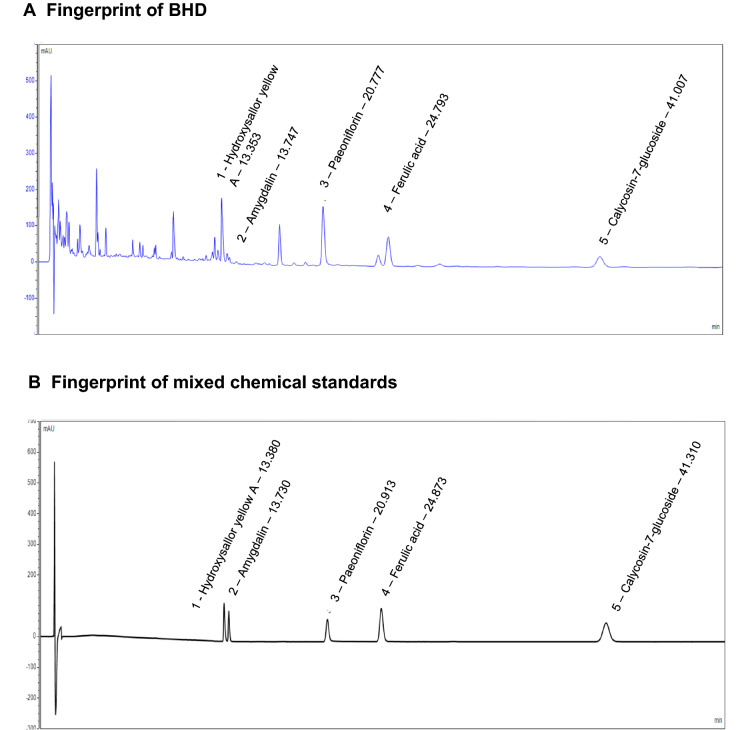
Representative HPLC chromatograms of BHD and mix standards. **A** HPLC chromatograms of BHD and **B** mixed chemical standards at 227 nm. Standards were: 1. Hydroxysafflor yellow A; 2. Amygdalin; 3. Paeoniflorin; 4. Ferulic Acid; 5. Calycosin-7-glucoside

We first performed HPLC study for quality control of BHD granules. We optimized the chromatographic conditions and obtained a well-separated chromatogram for the fingerprint of BHD (Fig. 1). The structures and retention time of those compounds are summarized in Additional file 1: Table S2. Five standard compounds were confirmed in BHD, including hydroxysafflor yellow A, amygdalin, paeoniflorin, ferulic acid and calycosin-7-glucoside. Thus, we used these compounds for quality control which are identified from *Astragali Radix*, *Angelicae Sinensis Radix Tail*, *Paeoniae Radix Rubra*, *Chuanxiong Rhizoma*, *Persicae Semen* and *Carthami Flos*.

We quantitatively analyzed the concentrations of hydroxysafflor yellow A, amygdalin, paeoniflorin, ferulic acid and calycosin-7-glucoside in BHD. As shown in Additional file 1: Table S3, the standard curves of the five chemical markers had good linearity with correlation coefficients (r)  >  0.999. Relative Standard Deviation (RSD) of peak areas for each compound was lower than 1.33% for stability assay. These results indicate that the HPLC method has good sensitivity, accuracy and stability. We subsequently used this method to determine the concentrations of five standard compounds in BHD samples (Additional file 1: Table S3). The contents of hydroxysafflor yellow A, amygdalin, paeoniflorin, ferulic acid and calycosin-7-glucoside were 1.601, 0.717, 7.635, 0.263 and 0.238 mg/g in BHD, respectively.

### BHD treatment reduces urinary protein excretion and improves renal pathology in STZ-induced diabetic nephropathy mice

**Fig. 2:**
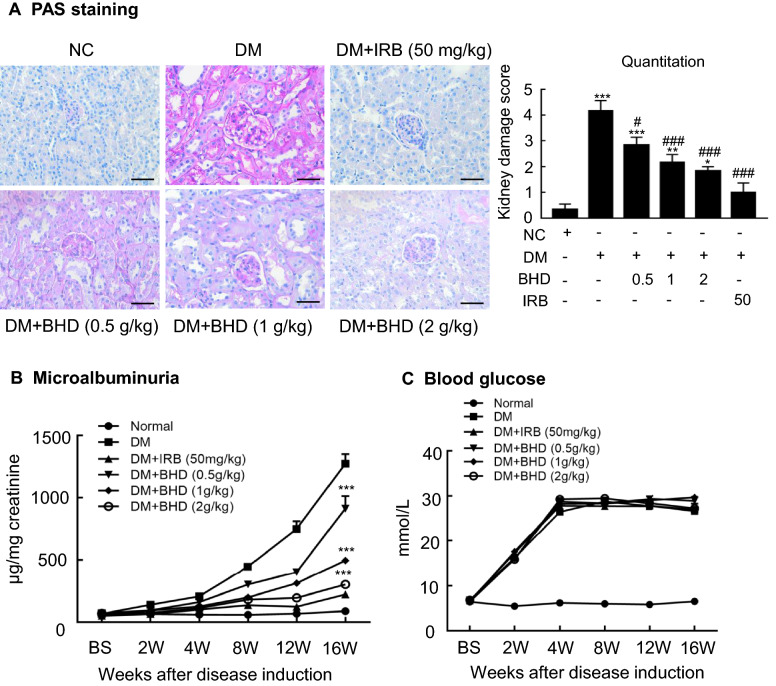
BHD restores renal function and alleviates kidney injury in STZ-induced diabetic mice. **A** Periodic acid Schiff (PAS) staining and score. Mesangial matrix expansion in glomeruli and the thickening of the glomerular basement membrane were observed. Scale bar: 50 µm. **B** Microalbuminuria, and **C** Blood glucose levels in diabetic mice over 16 weeks. DM, diabetes mellitus. Irbesartan (IRB) was taken as a positive control. Data represent the means  ±  SEM for groups of six animals. *P  <  0.05, **P  <  0.01, ***P  <  0.001 versus normal mice. ^#^P  <  0.05, ^##^P  <  0.01, ^###^P  <  0.001 versus DM mice

We evaluated the renoprotective effects of BHD (0.5, 1 and 2 g/kg/day) on STZ-induced diabetic nephropathy mice in which irbesartan was taken as a positive control drug. Periodic acid-schiff (PAS) staining revealed the stromal hyperplasia and thickened glomerular basement membrane in the sections of kidney tissues of the STZ-induced diabetic mice. Notably, BHD treatment dose-dependently reduced mesangial matrix expansion of glomeruli and the thickening of the glomerular basement membrane (Fig. 2A). Meanwhile, BHD treatment dose-dependently reduced urinary protein excretion level (Fig. 2B) without affecting blood glucose level (Fig. 2C). These results suggest that BHD has direct renoprotective effects in the diabetic nephropathy mice.

### BHD treatment protects against renal fibrosis and inflammation in STZ-induced diabetic nephropathy mice

**Fig. 3:**
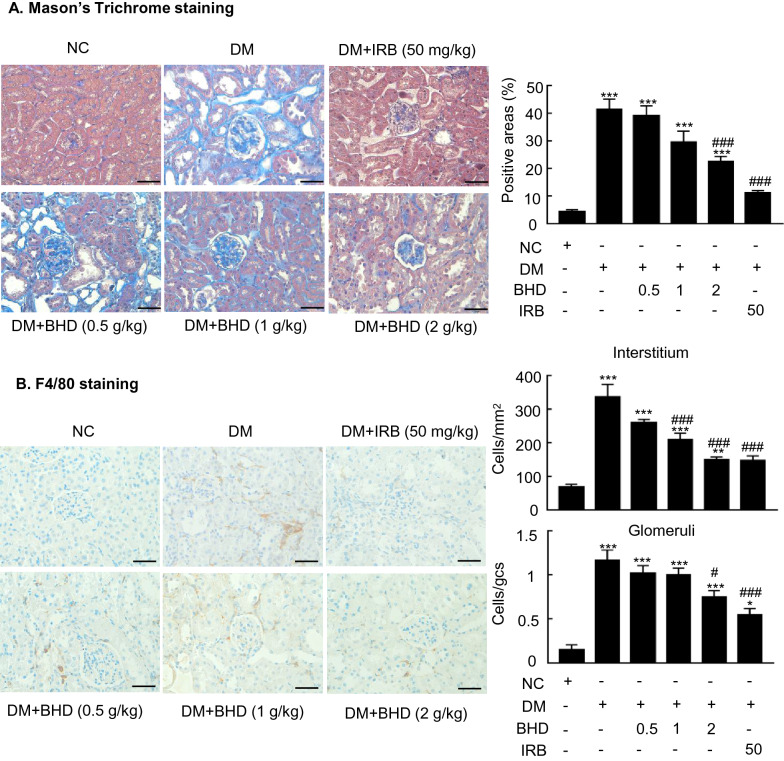
BHD reduces renal fibrosis and inflammation in STZ-induced diabetic mice. **A** Masson’s Trichrome staining and semi-quantification. Extracellular matrix deposition is shown in blue. **B** Immunohistochemistry of F4/80-positive cells showing macrophage infiltration in glomeruli and tubulointerstitium. DM, diabetes mellitus. Irbesartan (IRB) was taken as a positive control. Data represent the means  ±  SEM for groups of six animals. Scale bar: 50 µm. *P  <  0.05, **P  <  0.01, ***P  <  0.001 versus normal mice. ^#^P  <  0.05, ^##^P  <  0.01, ^###^P  <  0.001 versus DM mice

**Fig. 4:**
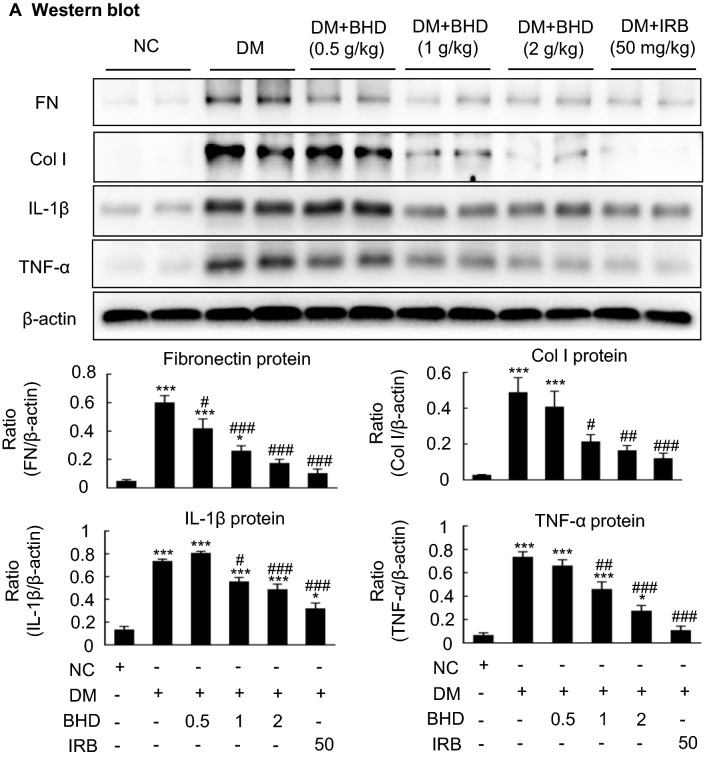
BHD reduces renal fibrotic and inflammatory factors in STZ-induced diabetic mice. **A** Western blot and quantitative analysis. BHD reduced the expression of fibronectin (FN), collagen I (Col I), interleukin-1β (IL-1β) and TNF-α compared to that in DM. DM, diabetes mellitus. Irbesartan (IRB) was taken as a positive control. Data represent the means  ±  SEM for groups of six animals. *P  <  0.05, **P  <  0.01, ***P  <  0.001 versus normal mice. ^#^P  <  0.05, ^##^P  <  0.01, ^###^P  <  0.001 versus DM mice

**Fig. 5:**
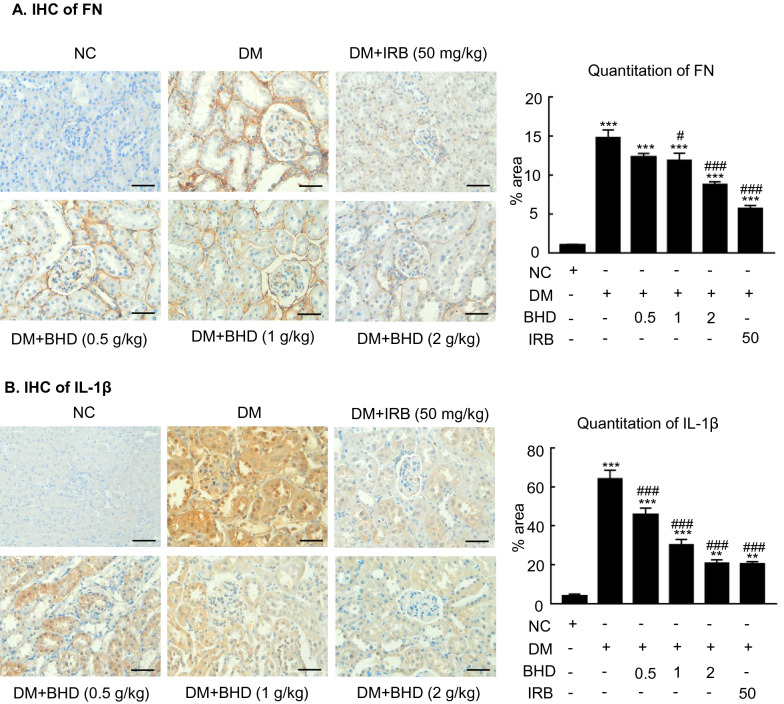
BHD reduces renal fibronectin and IL-1β in STZ-induced diabetic mice. **A** Immunohistochemistry of the fibronectin (FN) and **B** interleukin-1β (IL-1β). DM, diabetes mellitus. Irbesartan (IRB) was taken as a positive control. Data represent the means  ±  SEM for groups of six animals. Scale bar: 50 µm. *P  <  0.05, **P  <  0.01, ***P  <  0.001 versus normal mice. ^#^P  <  0.05, ^##^P  <  0.01, ^###^P  <  0.001 versus DM mice

Renal fibrosis and inflammation are two major pathological features of diabetic nephropathy [10]. We then examined the renoprotective effects of BHD on reducing renal fibrosis and inflammation in the STZ-induced diabetic mice. First, Masson’s Trichrome staining and semi-quantification revealed the renal fibrosis in the diabetic mice. BHD treatment reduced the renal extracellular collagen formation in a dose-dependent manner (Fig. 3A). Besides, immunohistochemistry showed that F4/80-positive macrophages were significantly increased in the diabetic renal tissues. BHD treatment dose-dependently decreased the positive staining cells, indicating the anti-inflammatory effects (Fig. 3B). Furthermore, western blot analysis showed that BHD treatment dose-dependently downregulated the expression of fibronectin, collagen I, TNF-α and IL-1β, indicating its anti-inflammatory and anti-fibrotic effects (Fig. 4). Immunohistochemistry studies yielded consistent results to western blot data, showing that the up-regulation of fibronectin and IL-1β in diabetic kidney sections were inhibited by the BHD treatment dose-dependently (Fig. 5A, B). BHD had similar effects to irbesartan, a positive control drug. Therefore, BHD could attenuate renal fibrosis and inflammation in the STZ-induced diabetic nephropathy mice.

### BHD inhibits TGF-β1/Smad3, NF-κB and Arkadia and restores Smad7 in renal tissues of STZ-induced diabetic nephropathy mice

**Fig. 6:**
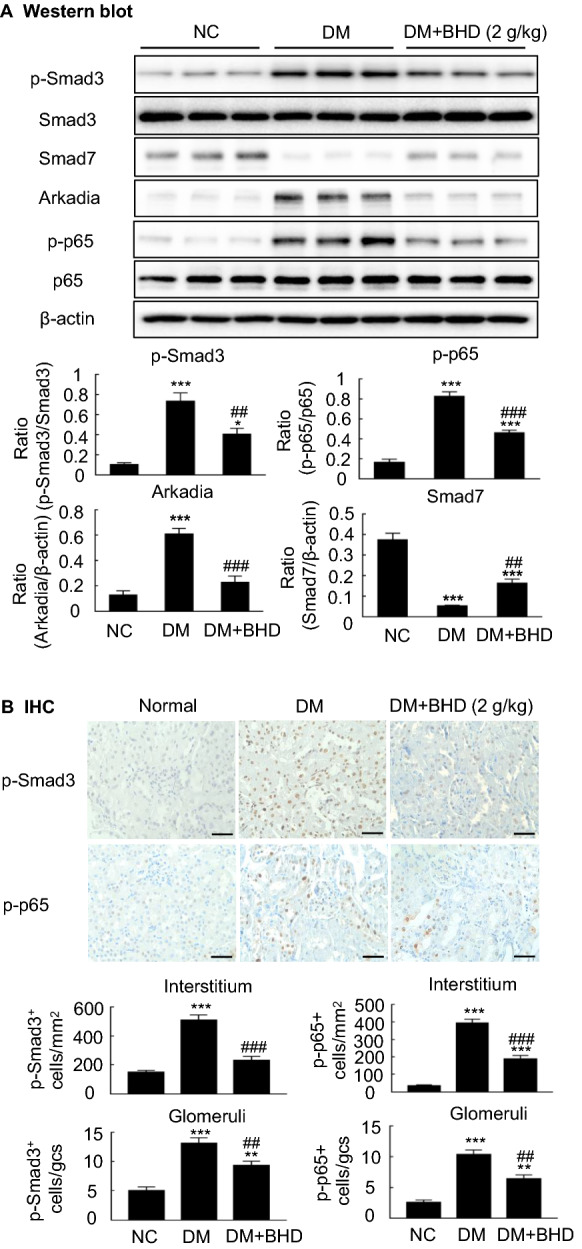
The underlying mechanisms by which BHD protects against diabetic nephropathy in vivo. **A** Western blot and quantitative analysis of phosphorylated Smad3, p65, Smad7 and Arkadia. **B** Immunohistochemical staining and quantitative analysis of phosphorylated Smad3 and p65 nuclear translocation in different groups. Scale bar: 50 µm. DM, diabetes mellitus. Data represent the means  ±  SEM for groups of six animals. *P  <  0.05, **P  <  0.01, ***P  <  0.001 versus normal mice. ^#^P  <  0.05, ^##^P  <  0.01, ^###^P  <  0.001 versus DM mice

TGF-β plays a critical role in the pathological progression of diabetic nephropathy [11]. TGF-β not only induces Smad3 phosphorylation but also enhances the Smad3-dependent Smad7 degradation by stimulating the Arkadia-mediated ubiquitin–proteasome degradation pathway [26–28]. Our previous study indicates that the depletion of Smad7 promotes the activation of NF-κB signaling in diabetic kidney [10]. Thus, we tested the hypothesis that the anti-fibrotic and anti-inflammatory effects of BHD might be attributed to blocking the Smad3 phosphorylation and Arkadia, subsequently increasing renal Smad7 and inhibiting NF-κB signaling in the STZ-induced diabetic nephropathy mice. The results were shown in Fig. 6. Western blot analysis and immunohistochemistry revealed the increased expressions of the phosphorylated Smad3 (p-Smad3) and the reduction of Smad7 in the renal tissues of diabetic nephropathy mice, which was companied with the upregulated expressions of E3-ligase Arkadia and phosphorylated NF-κB/p65 (p-p65) in the renal tissues. After diabetic nephropathy mice were treated with BHD (2 g/kg), the expressions of p-Smad3, Arkadia, p-p65 were significantly down-regulated whereas the expression of Smad7 was upregulated. Those results suggest that BHD could suppress the TGF-β1/Smad3 and NF-κB signaling pathways, while inhibiting Arkadia expression and restoring renal Smad7 in diabetic mice.

### BHD suppresses TGF-β1/Smad3-mediated fibrosis and NF-κB-driven inflammation, while suppressing Arkadia and restoring Smad7 in cultured mouse mesangial cells (MCs) in vitro

**Fig. 7:**
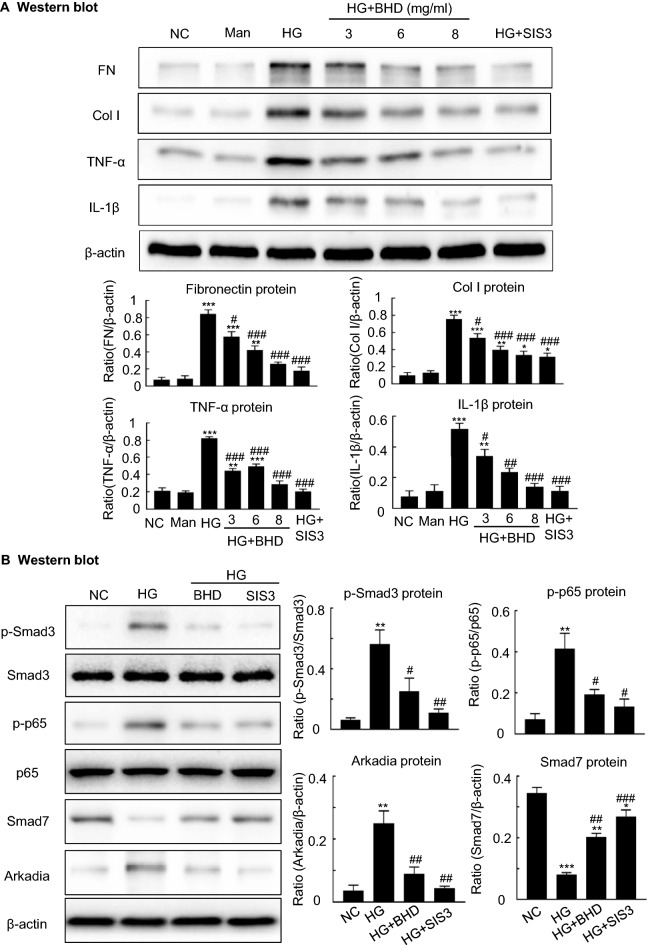
The underlying mechanisms by which BHD reduces high glucose-induced fibrosis and inflammation in vitro. **A** Western blot and quantitative data for fibronectin (FN), collagen I (Col I), interleukin-1β (IL-1β) and TNF-α. **B** Western blot and quantitative data for phosphorylated Smad3, phosphorylated p65, Smad7 and Arkadia. Man, mannitol (osmolality control). HG, high glucose; SIS3, a specific Smad3 inhibitor, 2 μM. Data represent the means  ±  SEM from 3–4 independent experiments. *P  <  0.05, **P  <  0.01, ***P  <  0.001 versus normal control. ^#^P  <  0.05, ^##^P  <  0.01, ^###^P  <  0.001 versus high glucose

We further tested the BHD’s renoprotective effects and its underlying mechanisms by using in vitro cultured MCs. These cells were stimulated by the conditions of low glucose (5.5 mM) and high glucose (35 mM) with or without BHD treatment (3, 6 and 8 mg/ml) for 24 h. We detected the expressions of pro-inflammatory cytokines including TNF-α and IL-1β and fibrosis-related proteins fibronectin and collagen I in the cells. Figure 7A revealed that high glucose exposure increased the expressions of TNF-α, IL-1β, fibronectin and collagen I. The BHD treatment dose-dependently down-regulated the expressions of fibronectin, collagen I, TNF-α, and IL-1β, indicating the anti-fibrotic and anti-inflammatory effects, respectively. We then used the dosage of 8 mg/ml of BHD for the following mechanistic studies. Figure 7B showed that high glucose exposure remarkably increased the expressions of p-Smad3, p-p65 and Arkadia but decreased the expression of Smad7. Notably, the BHD treatment significantly inhibited the expression of p-Smad3, p-p65 and Arkadia but increased the level of Smad7, whose effects were similar to SIS3, a Smad3 inhibitor. Those results suggest that BHD could suppress Arkadia expression and restore Smad7, contributing to the inhibitions of the TGF-β1/Smad3-mediated fibrosis and NF-κB-driven inflammation in the MCs.

### Calycosin-7-glucoside (CG), an active compound from BHD, attenuates high glucose-induced fibrosis and inflammation via blocking TGF-β1/Smad3 and NF-κB signaling pathways in MCs

**Fig. 8:**
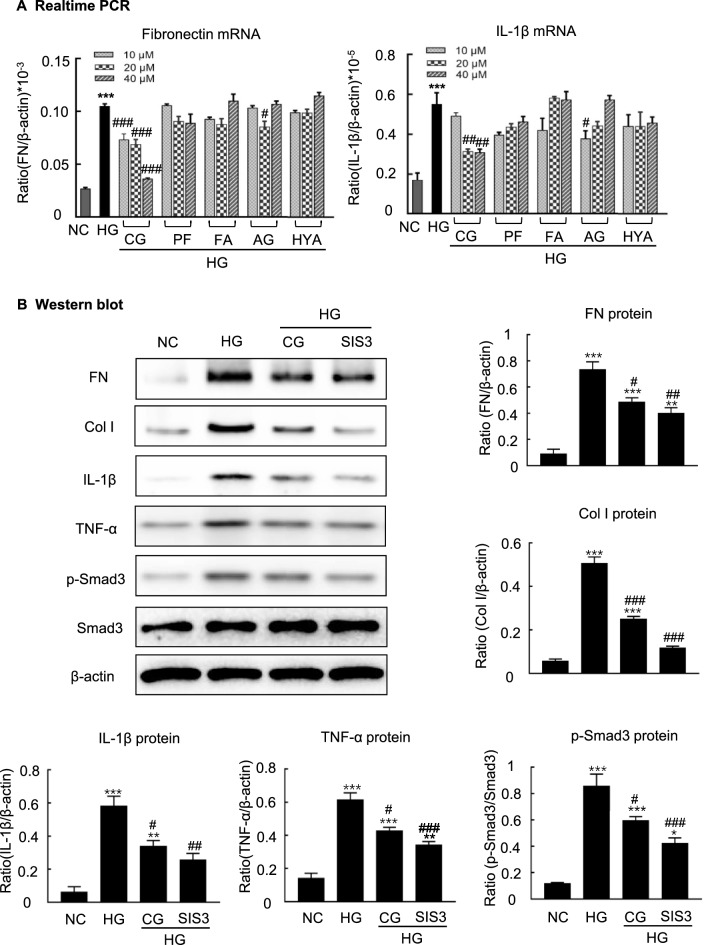
CG, an active compound from BHD, protects against high glucose-induced cell fibrosis and inflammation through regulating TGF-β1/Smad3 signaling. **A** Real-time PCR analysis of fibronectin and IL-1β mRNA levels for five compounds (10, 20 and 40 μM) under high glucose conditions in vitro. Five compounds: 1. calycosin-7-glucoside (CG); 2. paeoniflorin (PF); 3. ferulic acid (FA); 4. amygdalin (AG); 5. hydroxysafflor yellow A (HYA). **B** Western blot and quantitative data for fibronectin (FN), collagen I (Col I), interleukin-1β (IL-1β), TNF-α and phosphorylated Smad3. HG, high glucose; SIS3, a specific Smad3 inhibitor, 2 μM. CG: 40 μM. Data represent the means  ±  SEM from 3–4 independent experiments. *P  <  0.05, **P  <  0.01, ***P  <  0.001 versus normal control. ^#^P  <  0.05, ^##^P  <  0.01, ^###^P  <  0.001 versus high glucose

We then screened active compounds from BHD with anti-fibrotic and anti-inflammatory properties by using the in vitro cultured MCs under high glucose conditions. As showed in Additional file 1: Table S1, we selectively identified five compounds from the BHD sample. The cells were cultured in FBS-free medium for 24 h and then treated with these compounds respectively at the concentrations of 10, 20 and 40 μM under the exposures of low glucose (5.5 mM) or high glucose (35 mM) conditions for up to 24 h. We detected the expressions of fibronectin and IL-1β. Among five compounds, CG was found to be the most effective compound to inhibit the expression of fibronectin and IL-1β. RT-PCR results revealed that CG dose-dependently inhibited the expressions of fibronectin mRNA and IL-1β mRNA (Fig. 8A). Thus, CG was used as a representative active compound to further understand the anti-fibrotic and anti-inflammatory basis of BHD in the STZ-induced diabetic nephropathy mice.

**Fig. 9:**
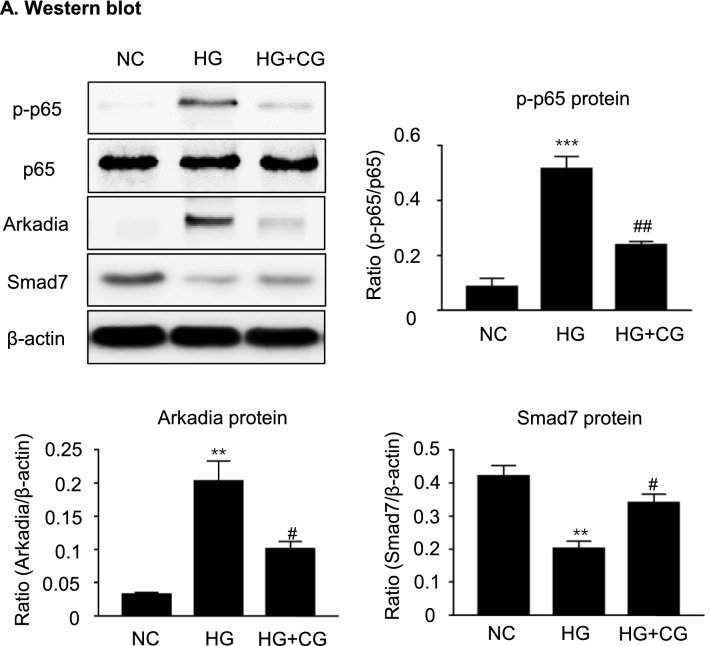
CG treatment reduces high glucose-induced phosphorylated p65, while suppressing Arkadia and restoring Smad7 in vitro. **A** Western blot and quantitative data for p-p65, Arkadia and Smad7. HG, high glucose. **P  <  0.01, ***P  <  0.001 versus normal control. ^#^P  <  0.05, ^##^P  <  0.01 versus high glucose

We used western blot analysis to further investigate the effects of CG on the expressions of p-Smad3, fibronectin, collagen I, TNF-α and IL-1β in the high glucose treated MCs. As showed in Fig. 8B, CG treatment downregulated the expression of p-Smad3 and inhibited the expressions of fibronectin, collagen I, TNF-α and IL-1β, which was consistent with the SIS3 treatment. Furthermore, we found that CG treatment also significantly reduced phosphorylated p65, while suppressing Arkadia expression and restoring Smad7 in high glucose treated MCs (Fig. 9). Taken together, CG could protect against high glucose-induced cell fibrosis and inflammation probably through regulating TGF-β1/Smad3 and NF-κB signaling pathways.

## Discussion

TGF-β1/Smad3 signaling pathway mediated renal fibrosis and NF-κB-driven renal inflammation are important pathological procedures to impair renal function in diabetic nephropathy [10, 13]. In the present study, we demonstrated that BHD has therapeutic effects against diabetic nephropathy by suppressing renal inflammation and fibrosis in vivo and in vitro. Mechanistically, BHD blocks TGF-β1/Smad3 signaling and inhibits renal inflammation by attenuating NF-κB signaling, while suppressing Arkadia expression and restoring renal Smad7. Furthermore, CG was found to be a representative active compound contributing to the anti-inflammatory and anti-fibrotic effects of BHD through inhibiting the TGF-β1/Smad3 and NF-κB signaling pathways. To the best of our knowledge, it is the first time to report that BHD and its active compound CG could protect against diabetic nephropathy via inhibiting TGF-β1/Smad3/NF-κB signaling.

BHD is a TCM formula composed of *Astragali Radix*, *Angelicae Sinensis Radix Tail*, *Paeoniae Radix Rubra*, *Chuanxiong Rhizoma*, *Persicae Semen*, *Carthami Flos* and *Pheretima.* Due to the multiple and complex chemical ingredients in BHD, we firstly performed a quality control study. According to Chinese Pharmacopoeia (2020 edition), calycosin-7-glucoside, ferulic acid, paeoniflorin, amygdalin and hydroxysafflor yellow A are active symbol chemical ingredients for *Astragali Radix*, *Angelicae Sinensis Radix Tail*, *Paeoniae Radix Rubra*, *Chuanxiong Rhizoma*, *Persicae Semen* and *Carthami Flos* respectively. Thus, we quantitatively detected these compounds in the preparations of BHD. We then conducted morphological studies and found that BHD treatment reduced mesangial matrix expansion of glomeruli and protected the glomerular basement membrane. Biochemical studies showed that BHD decreased the level of urinary protein excretion but without changing blood glucose levels. Thus, BHD has the direct renoprotective effects on the diabetic nephropathy mice.

We then investigated the underlying mechanisms of how BHD worked for improving renal functions and attenuating morphological changes. Diabetic nephropathy is defined as the appearance of chronic kidney disease in diabetes mellitus, accompanied by continuous elevation of urinary albumin excretion or a persistent reduction in estimated glomerular filtration rates [29]. The pathophysiological features of the STZ-induced diabetic nephropathy include the thickened basement membranes, mesangial expansion, hypertrophy, and glomerular epithelial cell (podocyte) loss in glomeruli and tubular interstitial fibrosis [30]. Mechanistically, TGF-β1/Smad3 signaling activations are crucial players in renal fibrosis and inflammation of diabetic nephropathy [9]. TGF-β1 was found to be significantly increased in the fibrotic kidneys of diabetic nephropathy [31]. TGF-β promotes phosphorylation of Smad3, affecting the promoter regions of fibrotic genes, e.g., collagens, CTGF, and stimulates the expressions of these genes. Interestingly, our previous study indicated that the activation of TGF-β1/Smad3 could be attributed to the loss of Smad7 signaling in diabetic nephropathy [10]. Smad7 is an inhibitory Smad which binds TGF-β type I receptor and blocks Smad3 phosphorylation. Smad7 also suppresses the phosphorylation of NF-κB/p65 by interacting with IκBα. The loss of Smad7 subsequently further enhances the activation of NF-κB/p65 [10, 26]. Thus, therapeutic strategies can be based on targeting the TGF-β1/Smad3 and NF-κB/p65 pathways to attenuate the progress of diabetic nephropathy. We logically investigated the effects of BHD on the TGF-β1/Smad3 and NF-κB/p65 signaling pathways in the STZ-induced diabetic nephropathy mice.

In previous studies, BHD was reported to inhibit TGF-β/Smads signaling-mediated cardiac fibrosis in the pressure overload-induced cardiac remodeling [32]. BHD also suppresses the inflammation by blocking the NF-κB signaling pathway in atherosclerosis rats [33]. Astragalus, a monarch herb of BHD, was found to protect against diabetic nephropathy via suppressing TGF‐β/Smad3 signaling in diabetic mice [34]. Thus, we tested the effects of BHD on the TGF-β1/Smad3 signaling pathway in the mice model of diabetic nephropathy. We found that BHD reduced the expressions of pro-inflammatory cytokines (TNF-α and IL-1β) and fibrosis-related proteins (fibronectin and collagen I), and inhibited the TGF-β1/Smad3 and NF-κB signaling pathway. Notably, Smad7 is a key protein to suppress the activation of TGF‐β/Smad3 and NF-κB signaling pathways in diabetic nephropathy [10]. Smad7 could be degraded by E3 ubiquitin-protein ligase Arkadia-mediated ubiquitination in diabetic kidneys [35]. As a result, we investigated the effects of BHD on the expression of Smad7. The results revealed that BHD treatment decreased Arkadia and restored renal Smad7 in diabetic mice. Consistently, our in vitro study also yielded similar results. Therefore, we speculate that BHD may protect against diabetic nephropathy probably by suppressing the Arkadia-dependent ubiquitin degradation of renal Smad7 and TGF-β/Smad3 signaling pathways. The exact underlying mechanisms remain to be further studied.

Previous studies suggest that *Astragali Radix*, *Angelicae Sinensis Radix Tail*, *Paeoniae Radix Rubra*, *Chuanxiong Rhizoma*, *Persicae Semen* and *Carthami Flos* could have renoprotective effects on diabetic nephropathy [36–38]. Astragalus root is a monarch herb in BHD, accounting for 57.2% of total weight. CG is a representative active ingredient from Astragalus root recorded in Chinese Pharmacopoeia (2020 edition). It was reported that CG could attenuate the inflammatory injury in vascular endothelial cells [39] and alleviate cerebral ischemia/reperfusion injury in rats [40]. Herein, CG treatment decreased high glucose-induced cell fibrosis and inflammation by reducing pro-inflammatory cytokines (TNF-α and IL-1β) and fibrosis-related proteins (fibronectin and collagen I), which was associated with the downregulation of TGF-β1/Smad3 and NF-κB/p65 pathways in MCs. These results suggest that CG could be a representative active compound contributing to the bioactivities of BHD in inhibiting the TGF-β1/Smad3 and NF-κB/p65 pathways and attenuating fibrosis and inflammation. However, whether CG directly interacts with the molecules within TGF-β1/Smad3 signaling or indirectly inhibiting TGF-β1/Smad3 signaling remains unclear. Additionally, the concentration of CG is 0.238 mg/g in granules. Orally administered BHD may also provide a sufficient concentration of CG to protect against diabetic nephropathy, which needs to be further studied. To our knowledge, it is the first time to verify the anti-inflammatory and anti-fibrotic bioactivities of CG, which might contribute to the renal protective effect of BHD on diabetic nephropathy.

Nevertheless, with the multiple ingredients of BHD, we could not ignore the effects of other active compounds individually or synergically to regulating the TGF-β1/Smad3 signaling pathways. For example, amygdalin, a chemical ingredient of *Persicae Semen*, down-regulated the expressions of FN and IL-1β mRNA in the high glucose treated MCs, indicating anti-fibrotic and anti-inflammatory effects. Besides, astragalus polysaccharides, ligustrazine and safflower yellow, also have renoprotective effects against diabetic nephropathy [41–43]. Astragalus polysaccharides could protect against STZ-induced diabetic rats through inhibiting the TGF-β/Smad signaling pathway [43]. Ligustrazine and safflower yellow improve renal function and reduce urine protein excretion in diabetic nephropathy patients [41, 42]. Thus, the anti-inflammation and anti-fibrosis for renoprotection could be attributed to the synergic effects of the multi-compounds from BHD, which should be further studied.

## Conclusion

BHD protects against STZ induced diabetic nephropathy by reducing renal inflammation and fibrosis. The underlying mechanisms could be related to inhibiting the TGF-β1/Smad3 and NF-κB signaling pathway, while suppressing Arkadia and restoring Smad7. In addition, CG, an active compound from BHD, attenuates high glucose-induced cell inflammation and fibrosis through blocking the TGF-β1/Smad3 and NF-κB/p65 signaling pathways. Thus, CG may be one of the active compounds contributing to the bioactivity of BHD to alleviate diabetic nephropathy.

## Supplementary Information

**Additional file 1: Table S1. **Individual medicinal materials of the BHD. **Table S2. **Representative compounds identified in BHD. **Table S3. **The validation of quantitative method and the contents of five chemical makers in BHD.

## Data Availability

Not applicable.

## Abbreviations

BHD: Buyang Huanwu Decoction
CG: Calycosin-7-glucoside
Col: Collagen
DN: Diabetic nephropathy
DM: Diabetes mellitus
FN: Fibronectin
HG: High glucose
IL-1β: Interleukin-1β
IRB: Irbesartan
NF-κB: Nuclear factor-κB
PAS: Periodic acid-schiff
TNF-α: Tumor necrosis factor-α
