# Supplementary material for: Buyang Huanwu Decoction protects against STZ-induced diabetic nephropathy by inhibiting TGF-β/Smad3 signaling-mediated renal fibrosis and inflammation
Source: Chin Med. 2021 Nov 14;16:118. doi: 10.1186/s13020-021-00531-1 (PMC8591830; doi:10.1186/s13020-021-00531-1)
Supplement: Supplementary file 1 — Additional file 1: Table S1. Individual medicinal materials of the BHD. Table S2. Representative compounds identified in BHD. Table S3. The validation of quantitative method and the contents of five chemical makers in BHD. [file 13020_2021_531_MOESM1_ESM.docx]

**Table 1 |** Individual medicinal materials of the BHD.

| **Material name** | **Ratio** | **Full scientific name** | **Major pharmacologically active constituents** |
| --- | --- | --- | --- |
| *Astragali Radix* | 57.2% | The dried root of *Astragalus mongolica（Fisch.）Bge* | Calycosin-7-glucoside |
| *Angelicae Sinensis Radix Tail* | 9.6% | The dried root of *Angelica sinensis* | Ferulic acid |
| *Paeoniae Radix Rubra* | 9.6% | The dried root tail of *Paeonia lactiflora Pall* | Paeoniflorin |
| *Chuanxiong Rhizoma* | 5.9% | The dried root of *Ligusticum chuanxiong Hort* | Ferulic acid |
| *Persicae Semen* | 5.9% | The dried mature seeds of *Prunus persica (L.) Batsch* | Amygdalin |
| *Carthami Flos* | 5.9% | The dried flower of *Carthamus tinctorius L* | Hydroxysafflor yellow A |
| *Pheretima* | 5.9% | *The dry body of Phheretima aspergillum (E.Perrier)* | N/A |

**Table 2 |** Representative compounds identified in BHD.

| **Peak no.** | **Retention time (min)** | **Formula** | **Molecular weight** | **Identification** | **Classification** | **Structure** |
| --- | --- | --- | --- | --- | --- | --- |
| 1 | 13.353 | C_27_H_32_O_16_ | 612.53 | Hydroxysallor yellow A | Flavone | 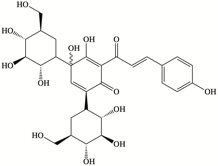 |
| 2 | 13.747 | C_20_H_27_NO_11_ | 457.43 | Amygdalin | Glycoside | 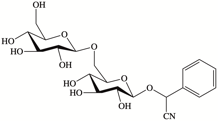 |
| 3 | 20.777 | C_23_H_28_O_11_ | 480.45 | Paeoniflorin | Glycoside | 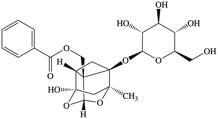 |
| 4 | 24.793 | C_10_H_10_O_4_ | 194.18 | Ferulic acid | Cinnamic acid | 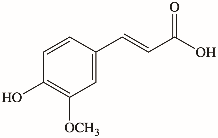 |
| 5 | 41.007 | C_22_H_22_O_10_ | 466.40 | Calycosin-7-glucoside | flavone | 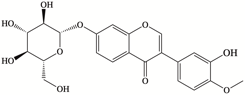 |

**Table 3 |** The validation of quantitative method and the contents of five chemical makers in BHD

| **Analyte** | **Linear Regression Data** | | **Relative Standard Deviation (%), n=3** | **Content**  **(mg/g NBF)** |
| --- | --- | --- | --- | --- |
|  | **Regression Curve** | **r** |  |  |
| Hydroxysallor yellow A | y=135.8502x-0.0536 | 0.99994 | 0.7589 | 1.601 |
| Amygdalin | y=28.4858x+0.0326 | 0.99985 | 1.1383 | 0.717 |
| Paeoniflorin | y=46.7572x-0.0868 | 0.99984 | 1.2246 | 7.635 |
| Ferulic acid | y=307.3947x-0.0578 | 0.9998 | 1.3269 | 0.263 |
| Calycosin-7-glucoside | y=682.0859x-0.1722 | 0.99993 | 0.8161 | 0.238 |

y=Analyte peak area (mAU*min); x=concentration of standards (mg/mL)
